# Endoscopic Ultrasonography-Guided Fine-Needle Aspiration for Duodenal Subepithelial Lesions Showing a Hypoechoic Mass on Endoscopic Ultrasound Imaging

**DOI:** 10.5152/tjg.2023.22696

**Published:** 2023-11-01

**Authors:** Kazuaki Akahoshi, Kazuya Akahoshi, Yuki Shiratsuchi, Shinichi Tamura, Kento Uemura, Reiichi Sashihara, Yoshihiro Ohishi, Kanako Inoue, Tadashi Koga, Hidenobu Koga

**Affiliations:** 1 Endoscopy Center, Aso Iizuka Hospital, Iizuka, Japan; 2 Department of Medical Research Promotion, Aso Iizuka Hospital, Iizuka, Japan; 3 Department of Pathology, Aso Iizuka Hospital, Iizuka, Japan; 4 Division of Central Laboratory, Aso Iizuka Hospital, Iizuka, Japan; 5 Department of Surgery, Aso Iizuka Hospital, Iizuka, Japan

**Keywords:** Endoscopic ultrasound-guided fine-needle aspiration, subepithelial lesion, duodenum, endoscopic ultrasonography, gastrointestinal stromal tumor

## Abstract

**Background/Aims:**

For duodenal subepithelial lesions showing a hypoechoic mass on endoscopic ultrasound imaging, the utility of endoscopic ultrasound-guided fine-needle aspiration and the frequency of histological types have not been the focus of previous literature. This study aimed to clarify this.

**Materials and Methods:**

This prospective observational study enrolled 22 consecutive patients who underwent endoscopic ultrasound-guided fine-needle aspiration for duodenal subepithelial lesions with hypoechoic mass on endoscopic ultrasound. Immunohistochemical analysis was performed for all endoscopic ultrasound-guided fine-needle aspiration and surgically resected specimens. The main outcome measures were the technical results of endoscopic ultrasound-guided fine-needle aspiration and the frequency of histological types of duodenal subepithelial lesions with hypoechoic mass.

**Results:**

Thirteen fine-needle aspiration specimens were obtained from the duodenal bulb and eight from the descending duodenal region. The puncture was not performed because of intervening vessels in one patient. The diagnostic rate was 81% (95% confidence interval: 58.1-94.6, 17/21 patients). In 12 patients receiving surgical resection (excluding one cancellation of endoscopic ultrasound-guided fine-needle aspiration), the diagnostic accuracy of endoscopic ultrasound-guided fine-needle aspiration was 75% (95% confidence interval: 42.8-94.5, 9/12 patients). No complications were observed. The histopathological diagnoses included 11 cases of gastrointestinal stromal tumor (50%), 2 cases of leiomyoma (9%), 2 cases of metastatic cancer (9%), 2 cases of benign inconclusive, and 1 case each of carcinoid, malignant lymphoma, leiomyosarcoma, gauzeoma, and aberrant pancreas (4.5% each). The frequency of malignant tumors in the duodenal subepithelial lesions with hypoechoic mass group was 73% (16/22 patients).

**Conclusions:**

Endoscopic ultrasound-guided fine-needle aspiration for duodenal subepithelial lesions with hypoechoic mass was safe and accurate. As duodenal subepithelial lesion with hypoechoic mass has a reasonably high possibility of containing malignant tumors, it is desirable to perform endoscopic ultrasound-guided fine-needle aspiration.

**Main Points**

- Duodenal subepithelial lesions showing a hypoechoic mass (DSELHM) on endoscopic ultrasound contain malignant tumors, such as gastrointestinal stromal tumor (GIST), and benign conditions, such as leiomyoma. The efficacy of endoscopic ultrasound-guided fine-needle aspiration (EUS-FNA) for DSELHM and the frequency of histologic types of DSELHM have not been thoroughly investigated.
- Our results suggest that EUS-FNA for DSELHMs is safe and accurate, and the frequency of malignant tumors, including GIST, in DSELHM is high.
- This study emphasizes that when encountering a DSELHM in daily clinical practice, performing EUS-FNA is desirable to obtain a conclusive histological diagnosis for selecting appropriate early treatment according to its high possibility of malignancy.

## Introduction

Duodenal subepithelial lesions (DSELs) are rare,^1^ although their detection rate is increasing with recent advances in endoscopy and observation technology. Benign DSELs include Brunner’s gland hyperplasia, lipoma, lymphangioma, leiomyoma, and ectopic pancreas, while malignant DSELs include gastrointestinal stromal tumor (GIST), metastatic cancer, malignant lymphoma, carcinoid, and leiomyosarcoma.^1-4^ Duodenal subepithelial lesions have a broad spectrum of histologic types, and corresponding management according to these types is needed. However, endoscopic and histologic diagnoses using conventional endoscopic biopsy are difficult because of the overlying normal mucosa.^1^

Endoscopic ultrasound (EUS) is the most important imaging modality for the differential diagnosis of gastrointestinal subepithelial lesions (SELs).^2,5,6^ The EUS can determine the origin of the gastrointestinal wall layer (i.e., within the submucosal layer, in continuity with the muscularis propria, or outside the wall), its content (i.e., liquid, fat, solid tumor, or blood vessel), and size of the gastrointestinal SEL.^7^ Therefore, EUS can provide a conclusive diagnosis of some lesions using echo findings only, including lipoma (high echoic mass), cystic lesion (anechoic mass), and varices. However, hypoechoic masses (HM) are also observed in malignant tumors, such as GIST, malignant lymphoma, metastatic cancer, neuroendocrine tumors, and SEL-like cancer, and benign conditions, such as leiomyoma, schwannoma, and aberrant pancreas.^2,7^ It is difficult to distinguish between these lesions using EUS findings alone^8,9^ and tissue acquisition is needed. Endoscopic ultrasound-guided fine-needle aspiration (EUS-FNA) is a reliable procedure for the conclusive immunohistochemical diagnosis of HMs in gastrointestinal SELs.^2,4,8^ Although histological features of HMs in gastric SELs using EUS-FNA have been reported,^10,11^ no studies have focused on duodenal subepithelial lesions with hypoechoic masses (DSELHMs). Herein, we prospectively evaluated 22 patients who underwent EUS-FNA with the detection of DSELHM by EUS at Aso Iizuka Hospital.

## Materials and Methods

### Patients

**Figure 1.**
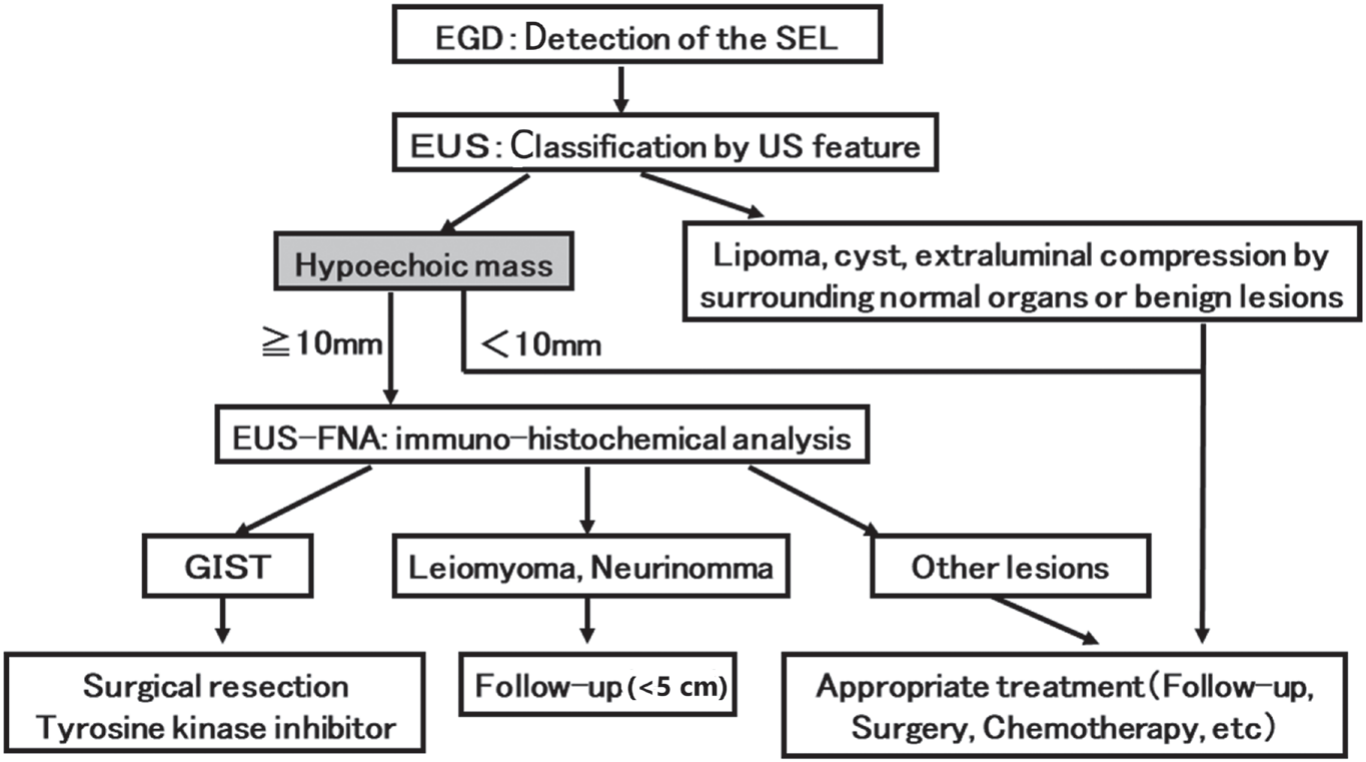
Our institutional diagnostic and therapeutic algorithm for gastrointestinal SELs using endoscopic ultrasound-guided fine-needle aspiration. Quoted and modified from references 11 and 12. EGD, esophagogastroduodenoscopy; EUS, endoscopic ultrasound; EUS-FNA, endoscopic ultrasound-guided fine-needle aspiration; GIST, gastrointestinal stromal tumor; SEL, subepithelial lesion.

Patients with DSELs were managed according to our institutional diagnostic and therapeutic algorithm for gastrointestinal SELs (Figure 1).^12^ Surgical resection was recommended for patients with histologically confirmed GIST (guided by immunohistochemical analysis of EUS-FNA specimen) according to the Japanese GIST guidelines^13^ after discussion with each patient. In our algorithm, EUS-FNA was performed for all DSELHMs >10 mm in diameter. This prospective study enrolled 22 consecutive patients (male:female, 15:7; mean age, 61.6 years) diagnosed with a DSELHM >10 mm in diameter by EUS who underwent EUS-FNA for differentiation of DSELHMs at our institution from October 2004 to June 2020. 

### Endoscopic Ultrasound and Endoscopic Ultrasound-Guided Fine-Needle Aspiration Procedures

**Figure 2.**
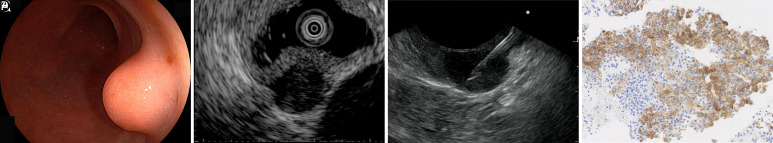
The EUS-FNA of a small duodenal GIST (surgically resected case) in a 48-year-old woman. (A) EGD showing a small SEL in the bulbus of the duodenum. (B) EUS showing an 11 mm diameter subepithelial hypoechoic solid tumor with continuity to the proper muscle layer. (C) Puncture of the small GIST under EUS guidance. (D) Immunohistochemical findings of the EUS-FNA specimen showing diffusely stained c-kit positive spindle and epithelioid tumor cells. EGD, esophagogastroduodenoscopy; EUS, endoscopic ultrasound; EUS-FNA, endoscopic ultrasound-guided fine-needle aspiration; GIST, gastrointestinal stromal tumor; SEL, subepithelial lesion.

Standard EUS was performed on an outpatient basis using a conventional radial scanner echoendoscope (GF-UM20: Olympus, Tokyo, Japan; or EG-530UR/EG-580UR: Fujifilm, Tokyo, Japan) or a 12 MHz ultrasound catheter probe (SP-702; Fujifilm) with the patient under conscious sedation. The EUS-FNA was performed on a 1-day inpatient basis using a convex array echoendoscope (PEF-708FA: Toshiba-Fujinon, Tokyo, Japan; or EG-530UT/EG-580UT: Fujifilm, Tokyo, Japan). The echoendoscope was connected to an ultrasound scanner (SSA-550A, Toshiba, Tokyo, Japan; SU-8000 or SU-1, Fujifilm). The FNA procedures were performed using 22G (NA-11J-KB, NA-200H, EZ shot2, EZ Shot 3Plus; Olympus, Tokyo) or 25G (Expect; Boston Scientific, Natick, Mass, USA) needles. All lesions underwent rapid pathological diagnosis (ROSE [rapid on-site evaluation]) by hemacolor staining (Auto-Hemacolor, Merck KGaAQ, Darmstadt, Germany). The EUS-FNA (Figure 2) was performed as previously described.^10,11^ In all cases, the diagnosis of DSELHM using EUS-FNA was made by histological diagnosis in the tissue obtained with EUS-FNA. The subsequent hematocrit was obtained on the first day after EUS-FNA, and patients were assessed for hematemesis or melena before discharge. 

### Immunohistochemical Analysis

The EUS-FNA and surgical resection specimens were fixed in 10% formaldehyde, and tissue blocks were embedded in paraffin. The sections were stained with hematoxylin and eosin. Immunoperoxidase staining was performed on the cell blocks and representative histological sections of the tumor using commercially available antibodies. The details of the antibodies used have been previously described.^10,11^ A tumor with a positive reaction to c-kit, CD34, or DOG1 was diagnosed as a GIST. A tumor with a negative reaction to c-kit, CD34, DOG1, and S-100, and a positive reaction to muscle actin was diagnosed as a myogenic tumor (leiomyoma). A tumor with a negative reaction to c-kit, CD34, DOG1, and muscle actin, and a positive reaction to S-100 was diagnosed as a neurogenic tumor (schwannoma).

## Assesment of Clinical Outcome

**Figure 3.**
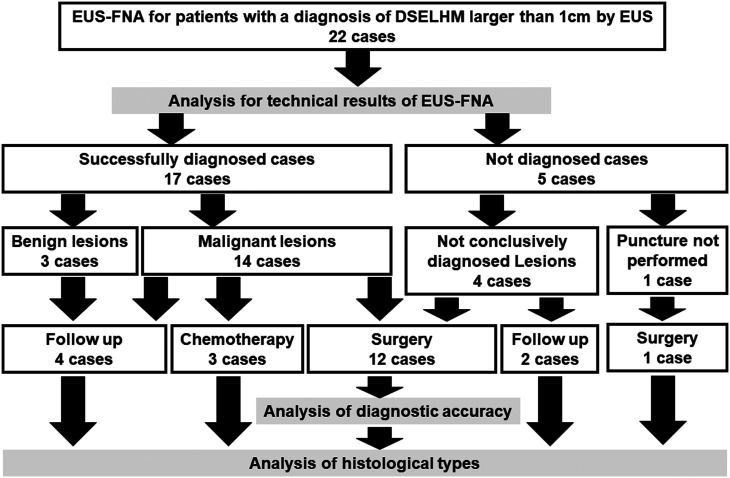
The flow diagram of this study. DSELHM, duodenal subepithelial lesions with hypoechoic mass; EUS, endoscopic ultrasound; EUS-FNA, endoscopic ultrasound-guided fine-needle aspiration.

The histological diagnostic rate, complications of EUS-FNA, and the frequency of the histological types of DSELHMs were evaluated in all 22 cases. The accuracy of the differential diagnosis of DSELHMs was calculated in 12 surgically resected patients with a diagnosis based on preoperative EUS-FNA (Figure 3).

### Statistical Analysis

95% confidence intervals (CIs) were appropriately calculated by statistical analysis using Stata version 15.0 (Stata Corp LLC, Tex, USA). 

### Ethical Approval

This study was performed at our institution and approved by the Ethics Committee of Aso Iizuka Hospital (registration no. 17129). This study is registered with the University Hospital Medical Information Network (UMIN) Clinical Trials Registry, number UMIN 000009972. Written informed consent was obtained from all the patients and the study performed in accordance with the Declaration of Helsinki.

## Results

The characteristics of the 22 patients who underwent EUS-FNA for DSELHMs are shown in Table 1. Lesions were present in the duodenal bulb in 13 patients and the descending part of the duodenum in 9 patients. The mean tumor size measured using EUS was 29.7 mm (range, 11-100 mm). 

The 22G FNA needles were solely used in 16 cases, 25G needles were solely used in 3 cases, and both were used in 2 cases. The mean number of passes in EUS-FNA was 2.57 (range, 1-5), excluding 1 discontinuation. The histological diagnosis rate of EUS-FNA was 81% (95% CI: 58.1-94.6, 17/21 patients), excluding 1 patient in whom the puncture was terminated (a safe puncture route could not be obtained because of blood vessels in the puncture route). No EUS-FNA procedure-related complications were noted. One patient who could not undergo EUS-FNA (due to an intervening blood vessel in the puncture route) underwent surgical resection because of clinical suspicion of GIST and the diagnosis was confirmed as GIST in the resected tissue. Two patients who underwent EUS-FNA without a conclusive histological diagnosis were surgically resected because of clinical suspicion of a GIST (1 patient had GIST, and the other had a gauzeoma). Two patients diagnosed with malignant tumors by EUS-FNA did not undergo resection. One patient with a carcinoid tumor was followed up at his request. The remaining patients had metastatic cancer and had received chemotherapy. Preoperative histological diagnosis was correctly obtained by EUS-FNA using immunohistochemical analysis in 9 of the 12 surgically resected cases (diagnostic accuracy: 75%, 95%; CI: 42.8-94.5), excluding the case with puncture cancellation (Table 1). The mean time interval between EUS-FNA and surgery was 8.3 weeks (range, 3-15 weeks).

The histological types of DSELHMs assessed using EUS-FNA or surgically resected specimens are shown in Table 2. The final histological diagnoses were 11 cases of GIST (50%), 2 cases of leiomyoma (9%), 2 cases of metastatic cancer (9%), 2 cases of benign inconclusive, and 1 case each of carcinoid, malignant lymphoma, leiomyosarcoma, gauzeoma, and an aberrant pancreas (4.5% each). The proportion of malignant tumors (GISTs, leiomyosarcomas, malignant lymphomas, metastatic cancers, and carcinoids) was 73% (16/22 patients).

## Discussion

Duodenal subepithelial lesions are rare; hence, the frequency of malignant disease in DSEL remains unknown. Currently, there are no guidelines or policies for the treatment of DSEL. Large DSELs and those associated with symptoms (e.g., bleeding and passage obstruction) are candidates for resection.^3,14^ By contrast, asymptomatic submucosal tumors ≤20 mm in diameter are treated on a case-by-case basis.^5,14^ In most cases, surgical resection is the treatment of choice when malignancy is suspected, but there are reports of less invasive endoscopic resection (ER) when the tumor size is small.^15,16^ Pre-treatment tissue diagnosis is important for selecting an appropriate treatment for DSEL. However, since DSELs are usually covered with normal epithelium, making a histological diagnosis using conventional endoscopic biopsy is difficult. These are often evaluated using EUS-FNA or other biopsy methods.^5^ The EUS-FNA is the standard tissue sampling method for SELs. This study reports our experience with EUS-FNA for DSELHM in 22 patients.

The diagnostic yields of EUS-FNA using various needle types to evaluate gastrointestinal SELs range from 52% to 87%,^11,17-21^ whereas the diagnostic accuracy of EUS-FNA in surgically resected patients ranges from 91% to 100%.^10,11,21,22^ De Moura et al^23^ also reported diagnostic accuracy of 73% for EUS-FNA in surgically resected patients with DSEL (n = 18). Comparatively, the diagnostic yield and accuracy of EUS-FNA in patients with DSELHM in this study were 81% and 75%, respectively. This study used conventional type 22- or 25-gauge needles to obtain histological samples. Recently available new needles, such as Franseen or Fork-tip type needles (fine-needle biopsy needles), could further improve the diagnostic rate (85%-89%) of all GI tract SELs.^24,25^ The incidence of EUS-FNA-related adverse events using 22-25-gauge needles for SELs was reported to be close to 0%,^10,26,27^ and no adverse events were observed in this study. Thus, EUS-FNA is an accurate and safe histological test for the definitive diagnosis of DSELHMs. 

The frequency of histological types of duodenal submucosal tumors has not been sufficiently investigated. Li et al^16^ performed ER in combination with ligation in 101 patients and reported the frequency of histological diagnosis using resected specimens. The frequency of benign disease was 86.1% (Brunner’s gland hyperplasia, 50.5%; lipoma, 18.8%; ectopic pancreas, 16.8%) and that of malignant disease was 13.9% (neuroendocrine neoplasm, 12.9%; GIST, 1%). El Chafic et al^28^ reported the frequency of histologic types using surgical pathology or EUS-FNA of 5 endosonographically suspected duodenal GI stromal tumors (HM on EUS). All of them were malignant tumors, including 3 neuroendocrine tumors (60%) and 1 (20%) each of GIST and metastatic tumors (20%). Differences in histological types in the earlier 2 studies were derived from the inclusion criteria (endoscopically resectable lesions vs. GIST-suspected lesions by EUS). Miettinen et al^29^ reported the frequency of immuno-histologic types of 190 duodenal mesenchymal tumors coded as leiomyomas, leiomyosarcomas, smooth muscle tumors, schwannomas, neurofibromas, nerve sheaths, or stromal tumors retrieved from the files of the Armed Forces Institute of Pathology and Haartman Institute of the University of Helsinki from 1970 to 1996. It showed malignant lesion as 94.2% (GIST 82.1%; leiomyosarcoma 2.6%; miscellaneous malignant tumors 9.5%) and benign lesions as 4.2% (leiomyoma 3.2%; schwannoma 1.1%). In this study, 73% of patients with DSELHM had malignant tumors. There were no cases of Brunner’s gland hyperplasia or lipomas in this study because Brunner’s gland hyperplasia may have been diagnosed by typical endoscopic and EUS findings (multiple cystic mass),^30^ and lipomas were excluded by typical EUS findings (hyperechoic mass). A previous study reported the histological typing of 90 gastric SELs <20 mm in diameter and HMs on EUS, with 47 cases (52%) of malignant SEL (44 GISTs, 1 glomus tumor, 1 SEL-like cancer, and 1 malignant lymphoma), 19 cases (21%) of benign SEL (14 leiomyomas, 4 ectopic pancreases, and 1 neurinoma), and 24 cases (27%) of indeterminate SEL.^10^ In the present study, although there were only a small number of DSELHM cases, there was a high proportion of malignant tumors in the DSELHMs (73%; 16/22 patients). Although the average tumor diameter was relatively large (29.7 mm), 7 cases <20 mm in diameter were included, 6 of which were GISTs. Our results indicate that DSELHM is frequently malignant regardless of size. Therefore, histological diagnosis of DSELHM is essential for the early diagnosis and treatment of these malignant diseases to improve prognosis.

The present study had a certain limitation. This study included a restricted sample size of 22 patients from a single center. Further large, multicenter, prospective observational studies with more cases are required to validate our findings.

In conclusion, DSELHMs have a reasonable possibility of being malignant tumors, including GISTs. The EUS-FNA should be considered for early diagnosis and treatment of DSELHMs. 

## Figures and Tables

**Table 1.** Characteristics of 22 Patients who Underwent EUS-FNA for DSELHM

Patient	Age/Sex	Site	EUS Findings of DSELHM			FNA-Diagnosis	Post-Surgical Diagnosis	Treatment
			Size (mm)	Continuity with mp	Appearance			
1	64/F	2nd por	43	Yes	Heterogeneous	GIST	GIST	Surgery
2	59/F	2nd por	30	Yes	Heterogeneous	FNA canceled	GIST	Surgery
3	47/M	Bulbus	15	Yes	Heterogeneous	GIST	GIST	Surgery
4	51/M	2nd por	25	Yes	Heterogeneous	BNC	GIST	Surgery
5	72/M	Bulbus	26	Yes	Homogeneous	GIST	GIST	Surgery
6	62/F	Bulbus	16	Yes	Homogeneous	GIST	GIST	Surgery
7	55/M	Bulbus	18	Yes	Heterogeneous	GIST	GIST	Surgery
8	48/F	Bulbus	11	Yes	Homogeneous	GIST	GIST	Surgery
9	82/M	2nd por	26	Yes	Heterogeneous	GIST	GIST	Surgery
10	67/M	Bulbus	12	Yes	Heterogeneous	Carcinoid	-	Patient requested follow-up
11	69/M	2nd por	18	Yes	Homogeneous	GIST	GIST	Surgery
12	51/M	2nd por	77	Yes	Heterogeneous	GIST	-	Chemotherapy
13	75/F	2nd por	32	Yes	Heterogeneous	Metastatic cancer	Metastatic cancer	Surgery
14	57/M	2nd por	100	Yes	Heterogeneous	Malignant lymphoma	-	Chemotherapy
15	45/F	Bulbus	30	Yes	Heterogeneous	GIST	Leiomyosarcoma	Surgery
16	76/M	Bulbus	20	Yes	Heterogeneous	Leiomyoma	-	Follow-up
17	60/F	Bulbus	30	Yes	Heterogeneous	Leiomyoma	-	Follow-up
18	58/M	2nd por	45	Yes	Heterogeneous	BNC	Gauzeoma	Surgery
19	59/M	Bulbus	14	Yes	Heterogeneous	BNC	-	Follow-up
20	70/M	Bulbus	20	Yes	Heterogeneous	BNC	-	Follow-up
21	87/M	Bulbus	20	Yes	Heterogeneous	Metastatic cancer	-	Chemotherapy
22	41/M	Bulbus	25	Yes	Heterogeneous	Aberrant pancreas	-	Follow-up

2nd por, second portion; BNC, benign, not conclusive; DSELHM, duodenal subepithelial lesions showing a hypoechoic mass on endoscopic ultrasound images; EUS, endoscopic ultrasound; EUS-FNA, endoscopic ultrasound-guided fine-needle aspiration; FNA, fine-needle aspiration; GIST, gastrointestinal stromal tumor; mp, muscularis propria.

**Table 2.** Frequency of Histological Types Assessed by EUS-FNA or Surgically Resected Specimens^*^ (n = 22)

Histologic diagnosis	n	%
**Malignant lesions**	**16**	**73**
GIST	11	50
Carcinoid	1	4.5
Malignant lymphoma	1	4.5
Metastatic cancer	2	9
Leiomyosarcoma	1	4.5
**Benign lesions**	**6**	**27**
Leiomyoma	2	9
Ectopic pancreas	1	4.5
Gauzeoma	1	4.5
Benign, not conclusive	2	9

EUS-FNA, endoscopic ultrasound-guided fine-needle aspiration; GIST, gastrointestinal stromal tumor.

^*^If there was a different diagnosis between that determined by EUS-FNA and the surgically resected specimen, the diagnosis using the surgically resected specimen was adopted. Bold values highlight the sum of malignant and benign tumors, respectively.
